# Clinicopathological characteristics and prognostic outcomes of young adult women (aged 18–30 years) with breast cancer in Ahmedabad, India: a single-centre, retrospective observational study

**DOI:** 10.1016/j.lansea.2025.100643

**Published:** 2025-07-28

**Authors:** Ajinkya Pawar, Poojitha Yalla, Mohit Sharma, Ketul Puj, Jebin Aaron, Vikas Warikoo, Abhijeet Salunke, Shashank Pandya

**Affiliations:** Department of Surgical Oncology, Gujarat Cancer and Research Institute, Ahmedabad, Gujarat, India

**Keywords:** Breast cancer, Young, Aggressive, High grade, LMIC

## Abstract

**Background:**

Breast cancer in young adult women is a rapidly growing group of cancer patients in India which needs to be addressed with urgency. Despite increasing global focus on breast cancer in young women, data from India remain scarce. Considering this gap, we undertook this study to analyse the clinicopathological characteristics and prognostic outcomes of young adult women (aged 18–30 years) with breast cancer in Ahmedabad, India.

**Methods:**

This was a retrospective observational study of a prospectively maintained database of 201 patients with breast cancer (aged 18–30 years) treated in a high-volume tertiary centre in Ahmedabad, India, from January 2015 to December 2020. Patients were followed up until June 2023. The demographic parameters, clinicopathological characteristics and survival of all patients were studied. Statistical analyses were done using SPSS and DATAtab.

**Findings:**

In this study 49.2% of cases were early breast cancers, 26.8% locally advanced, and 23.8% were metastatic. The proportion of aggressive cancers was higher with 38.8% hormone negative, 39.3% HER2-positive, 26.8% triple-negative and 50.8% grade 3. The median overall survival for all patients was 56 months (95% CI 28–84 months) and the 5-year overall survival was 48% (95% CI 40–56%). The multivariate analysis suggested that clinical stage, grade and luminal A status, significantly affected overall survival. The 5-year overall survival and disease-free survival of patients undergoing surgery were 65% (95% CI 57–74%) and 56% (95% CI 47–65%) respectively.

**Interpretation:**

The 5-year overall survival rate of 48% among young adult women with breast cancer included in this study is poor compared to the 77% observed in high-income countries in the western parts of the world. Adoption of appropriate and aggressive treatment strategies may enhance the outcomes in this age group of women with breast cancer.

**Funding:**

None.

**Research in context**

**Evidence before this study**

Breast cancer in young adult women is a rapidly growing group of cancer patients in India, which requires urgent attention. Previous research has indicated that a younger age at diagnosis is linked to more aggressive disease biology and worse prognoses for breast cancer. However, there is a paucity of research data on this special group of cancer patients in India.

**Added value of this study**

Analysing a cohort of 201 patients, this is one of the largest single-centre studies from India to focus exclusively on young adult women with breast cancer. This study offers a comprehensive clinicopathological and survival profile of women aged 18–30 years with breast cancer. We found that the 5-year overall survival rate of 48% in this group of patients is poor compared to the 77% observed in high-income countries in the western parts of the world.

**Implications of all the available evidence**

Understanding the clinicopathological characteristics of this underrepresented subgroup of patients may help in planning and adopting better treatment strategies. This may also enhance the outcomes of this special group of patients.

## Introduction

Breast cancer is the most common cancer and the leading cause of cancer-related mortality in India. It accounts for almost 26% of cancers in females with an incidence of 12.5 per 100 000 Indian population per year.^1^ Currently, India has the highest population in the world, with nearly 1.42 billion people, a median age of 28.5 years, and a sex ratio of 943 females per 1000 males. Also, the National Family Health Survey-5 (NFHS-5) survey revealed that 52% of India’s population is still younger than 30 years.^2^ This translates into a significant burden of breast cancer in young adult women in India. In the high-income countries in the western part of the world, breast cancer in young women is typically defined as occurring before the age of 35 years. However, the median age at diagnosis of breast cancer in India is 51 which is nearly a decade earlier than in high-income countries in the western part of the world and the cut-off of 35 years may not be appropriate.^3^ Furthermore, while breast cancer in young women (<35 years) accounts for less than 2% of all new cases in populations from high-income countries in western part of the world, Indian studies report a significantly higher incidence of 5–8% when the same cut-off of 35 years is used.^4^, ^5^, ^6^ We hypothesise that, an age cut off of 30 years may be more appropriate for describing this special group of patients.

Several studies have recognised breast cancers at a younger age as a distinct biologic entity with aggressive clinicopathological features and poor prognosis.^7^ In addition to cancer biology in young patients, a lack of awareness and screening contributes significantly to the higher stage at presentation.^8^ Also, the symptoms in young women are often mistaken for benign causes which further delays the diagnosis. Young breast cancers have higher risk of being associated with hereditary breast cancer syndromes, making genetic testing imperative to detect mutations.^9^ However, the rate of genetic testing in India is very low due to financial constraints.

There are gaps in treatment and a paucity of data on young breast cancer patients, especially in the young adult women of India. The aim of this study is to analyse the clinicopathological characteristics and prognostic outcomes of young adult women aged 18–30 years with breast cancer in an Indian tertiary oncology centre.

## Methods

**Fig. 1:**
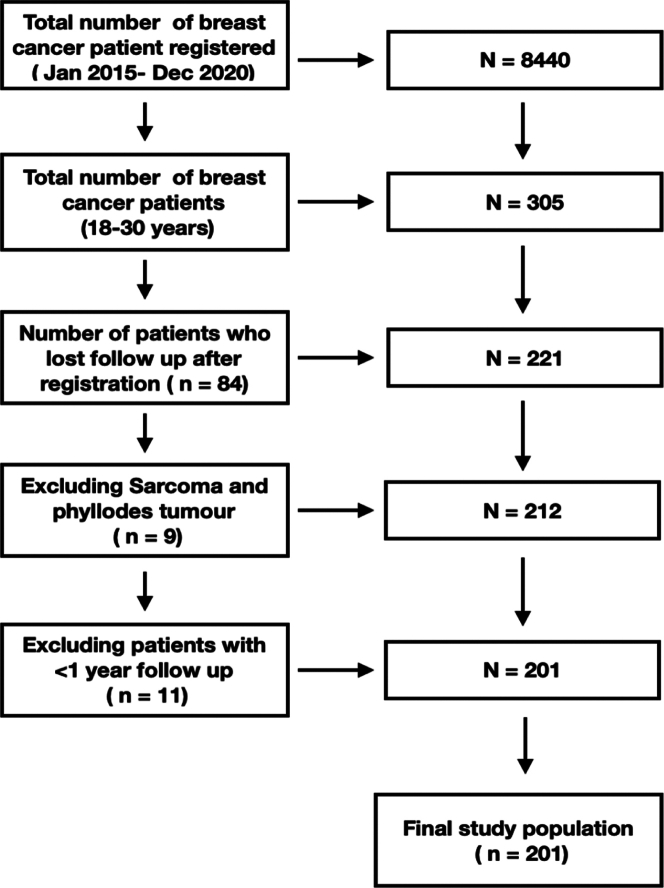
**Flow chart for selection of patients**.

This was a retrospective observational study of a prospectively maintained database of patients with breast cancer who registered at the Gujarat Cancer and Research Institute, Ahmedabad, Gujarat, from Jan 1, 2015, to Dec 31, 2020. A total of 8440 female patients of all ages with breast malignancies were registered. Patients with aged 18–30 years were selected (n = 305). Patients with sarcomas and phyllodes tumour were excluded from the study. Patients with less than one year of follow-up without any events of recurrence or death were also excluded. Finally, 201 patients in the age group 18–30 years with breast cancer were included in the study (Fig. 1). This study is reported in accordance with the STROBE guidelines for observational studies (Supplementary Information 1). Since this was a retrospective observational study, the requirement for ethics approval was waived by the institutional review board.

Patient details were obtained from the computer database and patient files. Follow-up was carried out on an outpatient basis and telephonically. The last follow up was conducted in June 2023. All patient characteristics were noted and documented. Diagnosis was confirmed by histopathology. Histological grading was performed according to the Elston and Ellis modification of the Scarff-Bloom-Richardson grading system (Nottingham grading). Immunohistochemistry and/or FISH testing was used to subtype cancer into Luminal A, Luminal B, HER2-enriched and triple-negative breast cancers (TNBC) according to oestrogen receptor (ER), progesterone receptor (PR), and human epidermal growth factor (HER2neu) receptor status. All patients underwent bilateral sonomammography before biopsy. Early Breast Cancer (EBC) patients were evaluated using chest X-ray and ultrasonography of abdomen and pelvis, while locally advanced breast cancer (LABC) patients were evaluated with contrast enhanced computed tomography (CECT) of thorax, abdomen, and pelvis with a bone scan. Positron Emission Tomography (PET) scan was done as indicated. Patients with LABC and some patients of EBC with high grade tumours received neoadjuvant chemotherapy (NACT). Upfront surgery was offered for EBC. Adjuvant chemotherapy, radiotherapy, and hormonal therapy were offered as indicated. Metastatic patients were offered palliative chemotherapy or hormonal therapy and/or palliative radiotherapy as indicated.

Clinicopathological characteristics and survival outcomes were studied. Disease-free survival (DFS) was defined as a period from date of diagnosis to date of recurrence or death. New primary or contralateral breast cancer was also considered as recurrence event. Overall survival (OS) was calculated from date of diagnosis to date of death and patients were censored at the last follow-up.

Statistical analysis was done using DATAtab and IBM SPSS version 29.0.2.0 statistical calculator. DFS and OS was calculated using Kaplan–Meier survival analysis. Univariate analysis was done using the log-rank test and multivariate analysis was done using Cox-proportional hazards model. A p-value less than 0.05 was considered as statistically significant.

### Role of the funding source

There was no funding source for this study.

## Results

A total of 201 patients in the age group 18–30 years who were diagnosed with breast cancer from January 2015 to December 2020 were included in the study. Fig. 1 describes the flow diagram for selection of patients. The number of patients in this age group constituted 2.35% of all patients with breast cancer. The median age at diagnosis was 29 years, and the median follow up was 35 months. Of these, 102 (50.7%) patients had right-sided, 95 (47.2%) patients had left-sided, and 4 (2%) patients had bilateral disease. 21 (10.4%) patients had a family history of cancers. Among these 21 patients, 15 (7.4%) patients had family history of *BRCA*-related cancers. Genetic counselling was done in 16 (7.9%) and genetic testing was done only in 6 patients (2.9%) of patients.

At the time of presentation, 99 (49.2%) of the cases were clinically early breast cancer, 54 (26.8%) were locally advanced breast cancer, and 48 (23.8%) were metastatic. On IHC subtyping, 68 (33.8%) were Luminal A type (ER and/or PR positive Her 2 negative), 55 (27.3%) were luminal B type (ER and/or PR positive Her 2 positive), 24 (11.9%) were HER2-enriched type (ER-, PR-negative and HER2-positive) and 54 (26.8%) were triple negative breast cancers. Of the 201 patients 191 (95%) had infiltrating ductal carcinoma-not otherwise specified (NOS), while the remaining 5% had other rare types of breast cancers. According to Nottingham grading system, 7 (3.5%) patients had grade 1, 92 (45.7%) had grade 2 and 102 (50.8%) had grade 3 disease (Table 1).

**Table 1:** Demographic and disease characteristics.

**Age (years) (n = 201)**	
Mean = 28 years, Median = 29 years	Range = 18–30 years
<25 (18–25)	32 (15.9%)
>25 (26–30)	169 (84.1%)
**Laterality (n = 201)**	
Right	102 (50.7%)
Left	95 (47.2%)
Bilateral	4 (2%)
**BMI (n = 201)**	
Mean = 22.4, Median = 21.6	
<18	28 (13.9%)
18–25	125 (62.1%)
25–30	38 (18.9%)
>30	10 (4.9%)
**Menarche (n = 201)**	
Median	13 years (9–16)
Mean	12.7 years
Early menarche (<12 years)	18 (8.9%)
**Marital status (n = 201)**	
Married	182 (90.5%)
Unmarried	19 (9.45%)
**Obstetric history (n = 201)**	
Nulliparous	36 (17.9%)
Parous	165 (82.1%)
≥2 Pregnancies	93 (46.2%)
1 Pregnancy	72 (35.8%)
Median age at First Pregnancy	23 years
1st Pregnancy within 10 years of menarche	101 (50.2%)
Nulliparous females who conceived after completion of treatment (n = 26)	5 (19.2%)
**Breast feeding**	
Yes	160 (79.6%)
No	41 (20.4%)
**Oral contraceptive pills**	
Yes	30 (14.9%)
No	171 (85.1%)
**Pregnancy associated breast cancer**	7 (3.4%)
**Family history (n = 201)**	
H/O Any cancer	21 (10.45%)
BRCA related cancer	15 (7.4%)
Genetic counselling	16 (7.9%)
Genetic testing	6 (2.9%)
**Clinical staging**	(n = 201)
**T stage**	
T1	5 (2.4%)
T2	90 (44.7%)
T3	70 (34.8%)
T4	36 (17.9%)
**N stage**	
N0	58 (28.8%)
N1	91 (45.2%)
N2	30 (14.9%)
N3	22 (10.9%)
**M stage**	
M0	153 (76.1%)
M1	48 (23.8%)
**Clinical stage**	
I	3 (1.5%)
II	96 (47.7%)
III	54 (26.8%)
IV	48 (23.8%)
**Clinical type at presentation (n = 201)**	
EBC	99 (49.2%)
LABC	54 (26.8%)
Metastatic	48 (23.8%)
**Luminal type (n = 201)**	
Luminal A	68 (33.8%)
Luminal B	55 (27.3%)
HER 2 enriched	24 (11.9%)
TNBC	54 (26.8%)
Hormone receptor positive	123 (61.1%)
Hormone receptor negative	78 (38.8%)
HER 2 positive	79 (39.3%)
HER 2 negative	122 (60.7%)
TNBC	54 (26.8%)
**Histology (n = 201)**	
Invasive ductal carcinoma	191 (95%)
Invasive lobular carcinoma	3 (1.5%)
Others	7 (3.5%)
**Grade (n = 201)**	
Grade 1	7 (3.5%)
Grade 2	92 (45.7%)
Grade 3	102 (50.8%)

TNBC, Triple negative breast cancer; EBC, Early breast cancer; LABC, Locally advanced breast cancer.

There were 153 patients in the locoregional group consisting of EBC and LABC patients. Of these, 144 patients (94.1%) underwent surgery. A total of 24 patients received NACT prior to surgery: 3 with EBC and 21 with LABC. Upfront surgery was performed in 120 patients. In the NACT group, 4 (16.7%) had pathological complete response (PCR) (all triple-negative), 15 (62.5%) had partial response, and 5 (20.8%) progressed on chemotherapy. Lymphovascular invasion (LVI), perineural invasion (PNI) and extranodal extension (ENE) were positive in 66 (45.8%), 12 (8.3%) and 41 (32.8%) patients respectively. A total of 126 (87.5%) patients undergoing surgery received chemotherapy (NACT or adjuvant), 104 (72.2%) received radiotherapy and 130 (90.2%) received hormonal therapy. Only 98 (68.1%) patients completed treatment while 46 (31.9%) patients did not complete the planned course of treatment. Of all the patients who underwent surgery, 50 patients (34.7%) had recurrence during the follow-up period: 40 had systemic recurrence while 10 had locoregional recurrence (Table 2).

**Table 2:** Details of patients undergoing surgery.

**Total locoregional disease**	N = 153
Surgery done	144 (94.1%)
Surgery omitted	9 (5.8%)
**Total patients undergoing surgery**	N = 144
**LVI (n = 144)**	
LVI positive	66 (45.8%)
LVI negative	78 (54.2%)
**PNI (n = 144)**	
PNI positive	12 (8.3%)
PNI negative	132 (91.7%)
**ENE (n = 125)**	
ENE positive	41 (32.8%)
ENE negative	84 (67.2%)
**Surgery (n = 144)**	
Mastectomy	130 (90.2%)
Breast conservation surgery	14 (9.7%)
Axillary lymph node dissection	131 (90.9%)
Sentinel lymph node biopsy	13 (9.1%)
**Neoadjuvant chemotherapy**	
Early breast cancer	3 (12.5)
Locally advanced breast cancer	21 (87.5)
**Response post-NACT (n = 24)**	
Partial response	15 (62.5%)
PCR post NACT	4 (16.7%)
Progressed	5 (20.8%)
**Treatment (n = 144)**	
No of patients who received chemotherapy (Neoadjuvant/Adjuvant)	126 (87.5%)
No of patients who received Radiotherapy	104 (72.2%)
No of patients who received Hormonal therapy	130 (90.2%)
Complete treatment	98 (68.1%)
Incomplete treatment	46 (31.9%)
No of patients who omitted chemotherapy (when indicated)	18 (12.5%)
No of patients who omitted Radiotherapy (when indicated)	40 (27.8%)
No of patients who omitted Hormonal therapy (when indicated)	14 (9.7%)
**Recurrence**	
Total recurrences	50 (34.7%)
Systemic recurrences	40 (27.8%)
Local recurrences	10 (6.9%)

LVI, Lymphovascular invasion; PNI, Perineural invasion; ENE, Extranodal extension; NACT, Neoadjuvant chemotherapy; PCR, Pathological complete response.

**Fig. 2:**
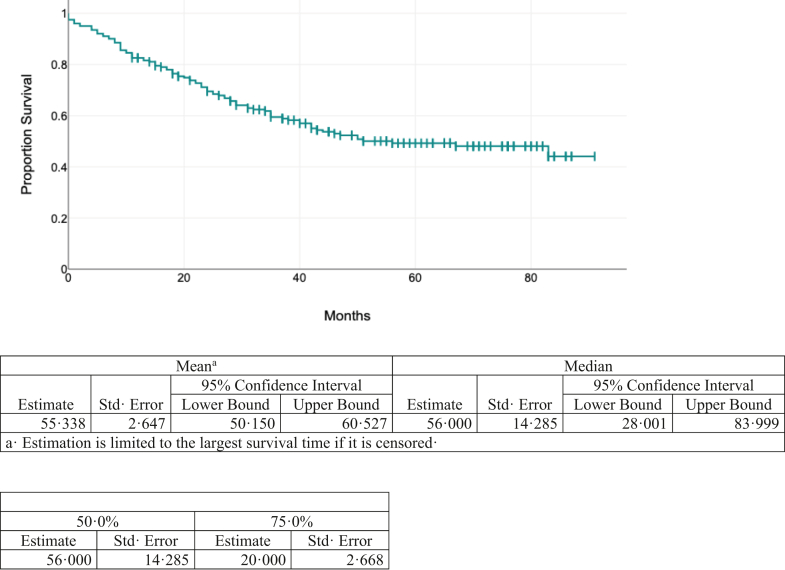
**Kaplan–Meier analysis of overall survival of all patients**.

**Fig. 3:**
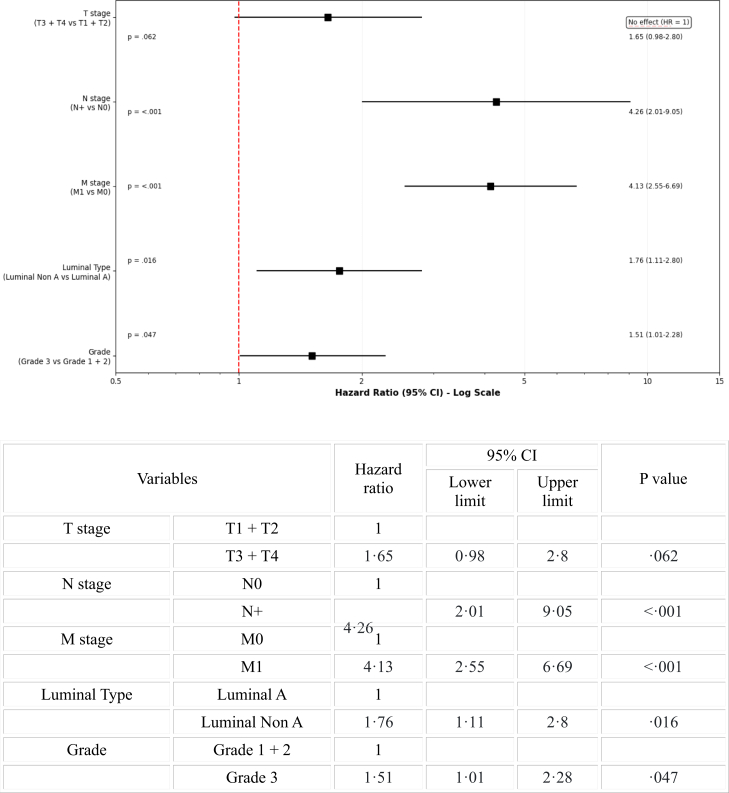
**Multivariate analysis of overall survival of all patients (Forest plot)**. CI – Confidence interval, HR – Hazard ratio

On Kaplan–Meier analysis (Fig. 2), median OS for all patients was 56 months (SE- 14.28, 95% CI 28–84 months, Mean OS 55.3 months, 75th percentile of 20 months) and 5-year OS was 48% (SE- 0.04, 95% CI 40–56%). On multivariate analysis with Cox proportional hazards model, N stage (N+, HR: 4.26, 95% CI 2.01–9.05, p < 0.001), M stage (M1, HR: 4.13, 95% CI 2.55–6.69, p < 0.001), Luminal type (Luminal Non-A, HR: 1.76, 95% CI 1.11–2.8, p = 0.016) and grade (grade 3, HR: 1.51, 95% CI 1.01–2.28, p = 0.047) were statistically significant (Fig. 3).

**Fig. 4:**
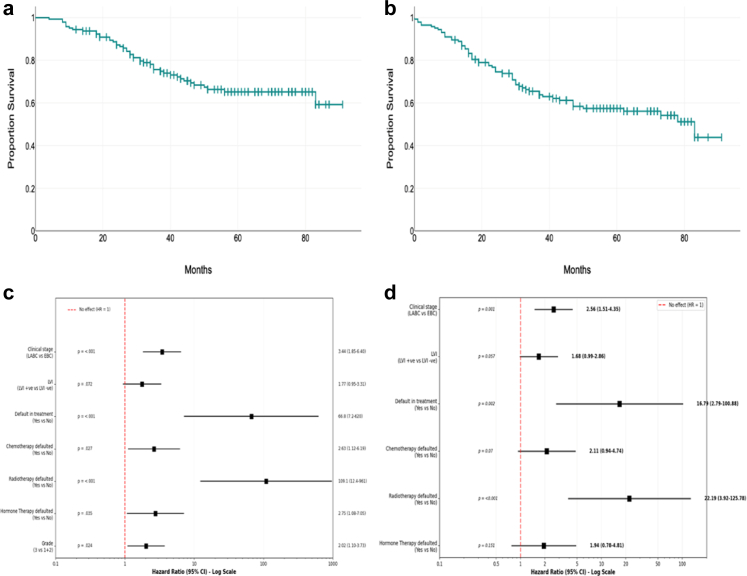
**Survival outcomes of patients undergoing surgery. a:** Kaplan–Meier analysis of Overall Survival of patients undergoing surgery. Median OS not reached. Mean OS 69 months (95% CI 63.7–74.3) 75th percentile 37 months. **b:** Kaplan–Meier analysis of Disease-Free Survival of patients undergoing surgery. Median DFS 83 months (95% CI 55.6–110.4) 75th percentile 24 months. **c:** Forest plot of Multivariate analysis of Overall Survival of patients undergoing surgery. **d:** Forest plot of multivariate analysis of Disease-Free Survival of patients undergoing surgery. OS, Overall Survival; DFS, Disease free survival; HR, Hazard ratio; EBC, Early breast cancer; LABC, Locally advanced breast cancer; LVI, Lymphovascular invasion; CI, Confidence interval.

On subgroup analysis of patients undergoing surgery (Fig. 4), 5-year DFS and OS were 56% (SE 0.04, 95% CI 47–65%) and 65% (SE 0.04, 95% CI 57–74%) respectively. Median DFS was 83 months (SE- 13.98, 95% CI 55.6–110 months, Mean OS 60.1 months, 75th percentile 24 months); median OS was not reached (mean OS 69 months, SE- 2.7, 95% CI 63.7–74.3 months, 75th percentile 37 months). Log-rank test and Cox regression analysis of DFS in patients who did not complete the planned course of treatment showed poor survival with HR of 1.9 (95% CI 1.14–3.18, p = 0.014) (Supplementary Information 2). On multivariate analysis of DFS, clinical stage (LABC, HR: 2.56, 95% CI 1.51–4.35, p = 0.001) and incomplete treatment (HR: 16.79, 95% CI 2.79–100.8, p = 0.002) were statistically significant. Log rank test and cox regression analysis of OS of patients who did not complete the planned course of treatment showed poor survival with HR of 2.9 (95% CI 1.61–5.2, p < 0.001) (Supplementary Information 3). On multivariate analysis of OS, clinical stage (LABC, HR: 3.44, 95% CI 1.85–6.40, p < 0.001), grade (grade 3, HR: 2.02, 95% CI 1.10–3.73, p = 0.024) and incomplete treatment (HR: 66.8, 95% CI 7.2–620, p < 0.001) were statistically significant.

## Discussion

Breast cancer in young women constitutes a significant cancer burden, especially in the transitioning and low- and middle-income countries (LMICs) such as India, where most of the population is young. The definition of breast cancer in young women is controversial with age threshold ranging from 30 years to 45 years, but 35 years is considered as a reasonable cut-off and adopted at the St Gallen international consensus.^10^^,^^11^ The incidence of breast cancer among young women (<35 years) in the western part of the world (especially in high-income countries), is around 2%, which matches the finding in our study (2.35%) when a cut-off of 30 years is used to define this group.^12^

Compared to breast cancer in older women, cancers arising in young women tend to be more aggressive with a higher grade of tumour and more LVI, PNI and ENE positivity.^13^ In our study as well, 50.5% had grade 3 disease, 45.8% had LVI, 8.3% had PNI and 32.8% had ENE positivity. Furthermore, hormone receptor-negative, HER2 positive, and triple-negative cancers, which are poor prognostic factors, are also more common in young women.^14^, ^15^, ^16^, ^17^, ^18^, ^19^, ^20^ These are comparable to our study group of young women, which includes hormone receptor-negative (38.8%), HER2-positive (39.3%) and triple-negative (26.8%) breast cancers. Our study also shows a higher proportion of T2 (44.7%) and T3 (34.8%) tumours, whereas the majority of patients from high-income countries in the western part of the world are in the T1 and T2 groups.^14^^,^^17^ This may be attributed to low levels of awareness, access to hospital and literacy in LMICs.^21^ The clinicopathological characteristics of disease in our study are comparable to the west when a lower cut-off of 30 years is used, this fact is underscored in Table 3.

**Table 3:** Comparison of clinicopathological and prognostic outcomes with the west.

Study	Age and Sample size	Stage at presentation	Tumour histopathology	Receptor status	Survival
Our study	18–30 years n = 201	I—1.5% II—47.5% III—26.8% IV—23.8% T1—2.4% T2—44.7% T3—34.8% T4—17.9% N0—28.8% N1—45.2% N2—14.9% N3—10.9%	Grade 1–3.5% Grade 2–45.7% Grade 3–50.8% PNI—8.3% LVI—45.8% ENE—32.8%	ER/PR + ve 61.1% ER/PR −ve 38.9% HER2 +ve 39.3% TNBC 26.8%	5-year OS—48% (stage I–IV) 5-year OS—66% (stage I–III) 5-year DFS—56% (stage I–III)
Liukkonen S et al.^14^ Finland 2011	≤35 years n = 212	T1—52% T2—41% T3—6% T4—1% N0—43% N1—35% N2—12% N3—8%	Grade 1–6% Grade 2–29% Grade 3–65%	ER/PR + ve 62% ER/PR −ve 38% HER2 +ve 34% TNBC 26%	5-year OS—80% 5 year DFS—70%
Kim et al.^15^ USA 2021	≤40 years n = 14 109	I—26.2% II—52% III—21%	Low/intermediate grade—40% High grade—54%	ER/PR+; Her2−: 31% ER/PR+; Her2+: 40% ER/PR−; Her2+: 7% TNBC: 19.5%	Compared with women aged between 40 and 60 years, women <40 years (hazard ratio 1.8, 95% CI 1.6 to 1.9) were more likely to die of breast cancer.
R A walker et al.^16^ UK 1996	<35 years n = 48	25–29 years n = 18 N +ve—69% N −ve—31% 30–34 years n = 30 N +ve—46% N −ve—54%	Grade 1–0% Grade 2–33% Grade 3–67% Grade 1–0% Grade 2–30% Grade 3–70%	ER +ve—44% PR +ve—33% Her2 +ve—22% ER +ve—57% PR +ve—37% Her2 +ve—20%	Not mentioned
Colleoni M et al.^17^ Italy 2001	<35 years n = 185	pT1—53.99% pT2—41.7% pT3/4–4.3% pN0—39.8% pN1—55.6% pN2/3–4.5%	Grade 1–8.2% Grade 2–29.9% Grade 3–61.9% LVI +ve—48.6%	ER + ve—61.2% PR + ve—50.9% Her2 +ve—39.7%	Not mentioned
Gonzalez-Angulo et al.^18^ USA 2005	≤35 years n = 452	I—29.7% II/II—65% IV—4.6%	Grade 1–2.7% Grade 2–27.9% Grade 3–69.38%	ER/PR +ve 61.6% ER/PR −ve 38.4% HER2 +ve 33.7%	5-year OS—77% 5-year RFS—48%
Martínez MT et al.^19^ Spain 2018	<35 years n = 258	T1—36.4% T2—43.4% T3—13.6% Missing—6.6% N +ve—46.9% N −ve—46.5% Missing—6.5%	Grade 1–11% Grade 2–27.5% Grade 3–36.4% Missing—25.2%	ER/PR + ve—68.5% ER/PR −ve—31.5% HER2 +ve—27.8% TNBC—13.5%	Increased percentage of relapse in younger women compared to control group. 46.4% vs. 24.7%, p = 0.0035
van der Hage JA et al.^20^ Netherlands 2011	<40 years Early breast cancers n = 549	T1—67% T2—32% T3—1% N +ve—37% N −ve—63%	Grade 1–15% Grade 2–32% Grade 3–53% LVI – 31%	ER/PR + ve 68% ER/PR −ve 32% HER2 +ve 26% TNBC 24%	10-year Overall survival Luminal A—94% Luminal B—78% Her2—93% TNBC—72%

ER, Estrogen receptor; PR, Progesterone receptor; OS, Overall survival; DFS, Disease free survival; RFS, Recurrence free survival; LVI, Lymphovascular invasion; PNI, Perineural invasion; TNBC, Triple negative breast cancer.

Family history and genetics play an important role in breast cancer, especially in young women. In a study of breast cancer under 30 years by Lalloo and colleagues, it was shown that mutations in *BRCA1*, *BRCA2*, and *p53* were found in half of patients with family history and 10% of patients without family history.^22^ In our study, 10.4% of patients had family history of cancer, genetic counselling was done in 7.9% and genetic testing was done only in 2.9% of patients. Ideally, all patients of breast cancer younger than 30 years should undergo genetic counselling and testing, but due to financial constraints, lack of facilities and awareness in LMICs, this is rarely feasible.

Management of breast cancer in young women requires a multimodal approach with special attention to psychosocial support, fertility and sexual health in these young women. Breast-conserving surgery positively impacts on the psychosocial and sexual well-being compared to mastectomy, and although the risk of local recurrence in young women (<35 years) is nine times higher than older women (>60 years), it does not affect overall survival.^23^^,^^24^ In a study by Gogia and colleagues, of 196 patients with operable breast cancer, 27% received NACT, 79% underwent modified radical mastectomy (MRM), and only 21% underwent breast conservation surgery (BCS). In a similar study by Das and colleagues, all patients with stage 2 and 3 disease underwent MRM, and 12 (out of 36) patients received NACT. In our study, of 153 women with locoregional disease, 144 underwent surgery; 16.6% of them received NACT and 9.7% of them underwent BCS.^4^^,^^8^ This lower rate of breast conservation surgery in India may be due to lack of awareness and concerns about disease recurrence.^25^

Fertility preservation remains a major unmet need in young women undergoing treatment for breast cancer in LMICs. In our study, around 18% (36 women) were nulliparous at the time of diagnosis and only (5 women)13.8% conceived after completion of treatment. While oocyte cryopreservation is an effective method for fertility preservation, its high cost and availability remain barriers. Ovarian suppression by gonadotropin releasing hormones (GnRH) analogues is a promising strategy proposed to preserve ovarian reserve during chemotherapy, and in hormone sensitive breast cancer, may improve survival.^26^^,^^27^ In our study, ovarian suppression was not offered to any patient.

Young age in breast cancer is associated with a lower 5-year DFS and OS compared to older women, even after adjusting for clinicopathological factors.^28^ Kim and colleagues showed that in women <40 years, breast cancer specific death was higher as compared to women in 40–60 years age group.^15^ Similarly, Bleyer and colleagues reported worse survival in women <35 years for all stages of breast cancer, with 5-year OS being 84% in stage 1–2, 47% in stage 3 disease.^29^ The 5-year overall survival in our study of 48% with a median OS of 56 months, which is lower compared to the high-income countries in the western part of the world (Table 3). This may reflect lower overall survival rates of breast cancers in LMICs due to delayed initiation of definitive management, inadequate treatment, and lack of access to latest technology.^30^ Further, in our study, only about 68% of patients with locoregional disease completed adequate treatment while 32% did not complete the planned course of treatment. The survival was better in women who underwent surgery and curative intent therapy (5-year OS 65%; 5-year DFS 56%). Discontinuation of chemotherapy and/or radiotherapy when indicated (i.e., treatment not completed) was associated with lower OS on multivariate analysis, underscoring the importance of treatment completion.

We acknowledge several limitations in our study. Since this is a retrospective observational study of a prospectively maintained database, it is susceptible to selection bias. Furthermore, as this research was conducted at a single high-volume centre, there is a possibility of institutional bias. Although this study represents one of the largest series of breast cancer in women aged <30 years in India, the sample size of 201 is relatively small.

Through this study, we highlighted the clinicopathological characteristics and prognostic outcomes of young adult women with breast cancer in Ahmedabad, India and attempted to address the gaps in data for this age group. This could help to devise better and aggressive management strategies for this group of patients with poor prognosis and thereby improve outcomes.

## Contributors

Conceptualisation: AP, MS, SP; Literature Search: JA, AS, VW; Data Extraction: PY, AP, JA; Data Review: AP, KP, VW, AS; Statistical Analysis: AS, KP, AP; Manuscript Writing: AP, JA, VW; Figures and Tables: AP, PY, JA; Manuscript Revision: AP, MS, SP.

## Data sharing statement

In view of the sensitive nature of the study population and institutional data protection policies, individual participant data, including data dictionaries and supporting documents, will not be made publicly available. At this time, there is no provision for external data sharing, as a formal data-sharing mechanism has not been established. However, individual deidentified data, other specified datasets, and any additional related documents will be made available by the corresponding author upon reasonable request, following the publication of this manuscript.

## Declaration of interests

We declare no competing interests.

## Appendix A Supplementary data

Supplementary Informations 1–3
